# Genotyping of β-Lactoglobulin gene by PCR-RFLP in Sahiwal and Tharparkar cattle breeds

**DOI:** 10.1186/1471-2156-7-31

**Published:** 2006-05-25

**Authors:** Satyanarayana Rachagani, Ishwar Dayal Gupta, Neelam Gupta, SC Gupta

**Affiliations:** 1 Animal Genetics and Breeding, Dairy Cattle Breeding Division, National Dairy Research Institute, Karnal, Haryana-132001, India; 2 DNA Fingerprinting Unit, National Bureau of Animal Genetic Resources, Baldy Bypass, Karnal, Haryana-132001, India

## Abstract

**Background:**

Improvement of efficiency and economic returns is an important goal in dairy farming, as in any agricultural enterprise. The primary goal of dairy industry has been to identify an efficient and economical way of increasing milk production and its constituents without increasing the size of the dairy herd. Selection of animals with desirable genotypes and mating them to produce the next generation has been the basis of livestock improvement and this would continue to remain the same in the coming years. The use of polymorphic genes as detectable molecular markers is a promising alternative to the current methods of trait selection once these genes are proven to be associated with traits of interest in animals. The point mutations in exon IV of bovine β-Lactoglobulin gene determine two allelic variants A and B. These variants were distinguished by Polymerase Chain Reaction and Restriction Fragment Length Polymorphism (PCR-RFLP) analysis in two indigenous *Bos indicus *breeds viz. Sahiwal and Tharparkar cattle. DNA samples (228 in Sahiwal and 86 in Tharparkar) were analyzed for allelic variants of β-Lactoglobulin gene. Polymorphism was detected by digestion of PCR amplified products with *Hae *III enzyme, and separation on 12% non-denaturing gels and resolved by silver staining.

**Results:**

The allele B of β-Lactoglobulin occurred at a higher frequency than the allele A in both Sahiwal and Tharparkar breeds. The genotypic frequencies of AA, AB, and BB in Sahiwal and Tharparkar breeds were 0.031, 0.276, 0.693 and 0.023, 0.733, 0.244 respectively. Frequencies of A and B alleles were 0.17 and 0.83, and 0.39 and 0.61 in Sahiwal and Tharparkar breeds respectively. The Chi-square test results (at one degree of freedom at one per cent level) revealed that the Tharparkar population was not in Hardy-Weinberg equilibrium as there was a continuous migration of animals in the herd studied, where as, the results are not significant for the Sahiwal population.

**Conclusion:**

Genotype frequencies of AA were the lowest compared to that of BB genotype in Sahiwal cattle while AB genotypes were more frequent in Tharparkar cattle. The frequency of A allele was found to be lower than that of B allele in both the breeds studied. These results further confirm that *Bos indicus *cattle are predominantly of β-Lactoglobulin B type than *Bos taurus *breeds.

## Background

Milk protein genetic polymorphism has received considerable research interest in recent years because of possible associations between milk protein genotypes and economically important traits in dairy cattle. Many research reports have indicated that certain milk protein variants may be associated with milk production [1-4], milk composition [5-15] and cheese production [5,6,12-14,16-20]. Therefore, milk protein genes could be useful as genetic markers for additional selection criteria in dairy cattle breeding. β-Lactoglobulin was the first protein in which polymorphism was detected. By paper electrophoresis two distinct bands of β-Lactoglobulin were observed, and named as β_1 _and β_2 _(A & B) [21]. Until now at least 12 variants are known for β-Lactoglobulin, out of which A and B variants are more frequent. β-Lactoglobulin is the major whey protein in milk of cows and other ruminants e.g. deer, bison and buffalo, and in some non-ruminants such as pigs, horses, dogs, dolphins and whales. However, it is not an endogenous part in human milk.

β-Lactoglobulin is amphiphatic and an extremely acid stable protein which exists at the normal pH of bovine milk as a dimer with a molecular weight of 36,000 Daltons. It is a single chain polypeptide of 18 kDa comprising of 162 amino acid residues. The complete amino acid sequence of β-Lactoglobulin has been reported and genetic variation in amino acids sequence has been identified [22]. The bovine β-Lactoglobulin A variant differs from B variant by two amino acids only i.e. aspartate-64 and valine-118. These amino acids are substituted by glycine and alanine respectively in the B variant. All the variants contain five cysteine residues, four of which are involved in forming intra-chain disulphide bridges. The biological functions of this protein are still not known. It could have a role in metabolism of phosphate in the mammary gland and the transport of retinol and fatty acids in the gut [12]. Sahiwal and Tharparkar cattle are the best dairy breeds in the Indian subcontinent. The major breeding tracts of Sahiwal lie in Montgomery district of Pakistan and in the Indian states of Punjab and Haryana. The color ranges from reddish brown to a more predominant red, with varying amounts of white on the neck, and the underline. In males, the color darkens towards the extremities, such as the head, legs and tail. The typical Tharparkar cattle are found in the areas of Umarkot, Naukot, Dhoro Naro, Chhor, Mithi, Islamkot and Khari Ghulam Shah of Pakistan and also in the adjoining Indian States of Rajasthan and Gujarat. The usual color of the cattle is white or gray. Tharparkar are of the lyre horned type of zebu cattle. The present study aims at genotyping the two *Bos indicus *breeds viz. Sahiwal and Tharparkar for β-Lactoglobulin gene.

## Results and discussion

**Figure 1:**
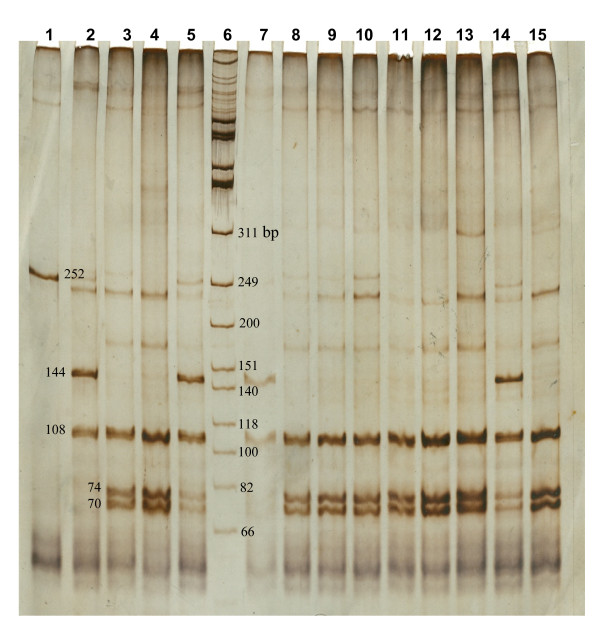
PCR-RFLP of β-lactoglobulin gene polymorphism using *Hae III *restriction endonuclease enzyme in Sahiwal cattle. Lane 1: PCR product, Lane 2 and 7: AA, Lane 3, 4, 8–13 and 15: BB, Lane 5 and 14: AB and Lane 6 ladder:Φ × 174 Hinf I digest.

The restriction digestion of 252 bp PCR product with *Hae *III enzyme revealed three genotypes AA, AB and BB (Figure 1). The fragment size of different genotypes is shown in Table 1. The genotypic frequencies of AA, AB, and BB were 0.031, 0.276, and 0.693 in Sahiwal, and 0.023, 0.733, and 0.244 in Tharparkar respectively. In Sahiwal, the genotype frequencies of BB were the highest while in Tharparkar cattle the AB genotypes were more frequent. These results also show that the chosen primers are adequate to amplify the sequence of β-Lactoglobulin gene exon IV in *Bos indicus *cattle. Gene frequencies of A and B alleles were 0.17 and 0.83 in Sahiwal, and 0.39 and 0.61 in Tharparkar respectively (Table 2). The frequency of A allele was found to be lower than that of the B allele in both the breeds studied, and in close agreement to the results of earlier workers in *Bos taurus *[23] and *Bos indicus *[24]. The genotyping results of Sahiwal and Tharparkar breeds are similar to those reported in Gyr, Nelore and Sindi cattle [25]. Polymorphism studies conducted in dual purpose Gyr and Nelore breeds showed higher frequencies of B allele than beef breeds [26]. However, AB genotype was more frequent in Tharparkar cattle. These results further confirm that *Bos indicus *cattle are predominantly of β-Lactoglobulin B type as compared to *Bos taurus *cattle. The Chi-square test results (at one degree of freedom at one per cent level) are presented in Table 2 revealing genetic equilibrium in Sahiwal cattle. In Tharparkar, the Chi-square test revealed that the Tharparkar population was not in Hardy-Weinberg equilibrium as there was a continuous migration of animals in the herd studied.

**Table 1:** Fragment size corresponding to different β-lactoglobulin genotypes after digestion of a 252 bp PCR product with *Hae *III restriction enzyme.

**Genotype**	**Fragment size after digestion with *Hae *III restriction enzyme**	**No. of genotypes**	
Uncut PCR Product	252 bp	**Sahiwal (n = 228)**	**Tharparkar (n = 86)**

A/A	144 bp and 108 bp	7	2
A/B	144 bp, 108 bp, 74 bp and 70 bp	63	63
B/B	108 bp, 74 bp and 70 bp	158	21

**Table 2:** Gene and genotypic frequencies of β-Lactoglobulin gene determined by PCR-RFLP in Sahiwal and Tharparkar breeds.

**Breed**	**No. of animals**	**Chi-square test **(one degree of freedom)	**Gene frequency**			**Genotype frequency**		
			
			**A**	**B**	**SD**	**AA**	**AB**	**BB**
Sahiwal	228	0.03^NS^	0.17	0.83	0.025	0.031	0.276	0.693
Tharparkar	86	25.0***	0.39	0.61	0.052	0.023	0.733	0.244

Note: NS: Not significant; *** P < 0.0001 (one degree of freedom)

## Conclusion

The frequencies of AA genotype was the lowest while that of BB genotype highest in Sahiwal cattle whereas the AB genotypes were more frequent in Tharparkar animals. Frequency of A allele was found to be lower than that of B allele in both the breeds studied.

## Methods

Blood samples were collected in vacutainers (Bacton-Dickinson vacutainer system) containing sodium EDTA as an anticoagulant from 228 Sahiwal and 86 Tharparkar cattle breeds maintained at National Dairy Research Institute, India. Genomic DNA was extracted using phenol chloroform method from 10 ml of whole blood [27] and semen [28]. The quality of DNA was checked on 0.6% agarose and quantity by UV spectrophotometer at A_260_/A_280 _nm. The samples having OD ratio between 1.7–1.9 were considered good, and used for polymerase chain reaction.

The sequences of primers [29] used for amplification of exon IV of β-Lactoglobulin gene containing polymorphic sites for A and B alleles were: 5'-GTC CTT GTG CTG GAC ACC GAC TAC A-3' (forward) and 5'CAG GAC ACC GGC TCC CGG TAT ATG A-3' (reverse). The PCR amplification reaction contained 100 ng DNA, 50 ng/μl of each primer, 1.5 mM of MgCl_2_, 100 μM of dNTPs, PCR buffer (10 mM KCl, 10 mM (NH_4_)_2_SO_4_, 20 mM Tris-HCl, 2 mM MgSO_4_, 0.1%TritonX-100, pH 8.8) and 0.7 units of Taq DNA polymerase. The fragment was amplified by hot start PCR, in which 2 μl of genomic DNA and 1.5 μl of Tris (20 mM) were placed in a PCR tube overlain with a thin layer of mineral oil. The PCR amplification was carried out in a programmable thermal cycler (MJ research) using the following program: denaturation at 95°C for 5 minutes, the temperature lowered to 85°C and PCR master mix added at the top of the mineral oil. The PCR was as follows: 3 minutes at 97°C, 1 minute at 60°C, 1 minute at 72°C followed by 34 cycles of 1 minute at 94°C, 1 minute at 60°C and 1 minute at 72°C and a final extension of 10 minutes at 72°C. The PCR products were loaded on 1.5% agarose to confirm the amplification of target region using Φ × 174 *Hinf *I digest as a marker. The restriction digestion of the PCR products were carried out with *Hae *III enzyme [29] with few modifications: the PCR products were subjected to digestion by restriction enzymes in a total volume of 25 μl. The reaction was set up with 5.00 μl of ddH20, 2.5 μl of restriction endonuclease buffer 2 (New England Biolabs), 3.6 units of Hae III and 17.0 μl of PCR product, and incubated at 37°C for about 3 hours. The reaction was stopped by adding 0.5 M EDTA to a final concentration of 10 mM. The restriction digested fragments were separated on 12% non-denaturing gels using PROTEAN II xi cell (Bio-rad, USA) at 4 v/cm for about 6 hours. The gels were stained by silver staining [30] and the three genotypes AA, AB and BB scored manually. Chi-squere test [31] was conducted to test the population for Hardy-Weinberg equilibrium.

## Authors' contributions

**RS **carried out molecular genetics studies, statistical analysis and drafted the manuscript.

**IDG **was responsible for conceived the study, concept, designed, coordinated the study and corrected the manuscript.

**NG and SCG **provided their laboratory facilities for carrying out experimental work.

All authors have read and approved the final manuscript
